# Neuroprotective Effect of 6-Paradol in Focal Cerebral Ischemia Involves the Attenuation of Neuroinflammatory Responses in Activated Microglia

**DOI:** 10.1371/journal.pone.0120203

**Published:** 2015-03-19

**Authors:** Bhakta Prasad Gaire, Oh Wook Kwon, Sung Hyuk Park, Kwang-Hoon Chun, Sun Yeou Kim, Dong Yun Shin, Ji Woong Choi

**Affiliations:** 1 Laboratory of Pharmacology, College of Pharmacy and Gachon Institute of Pharmaceutical Sciences, Gachon University, Incheon, Republic of Korea; 2 Laboratory of Pharmacognosy, College of Pharmacy, Gachon University, Incheon, Republic of Korea; 3 Laboratory of Medicinal Chemistry, College of Pharmacy, Gachon University, Incheon, Republic of Korea; 4 Laboratory of Biochemistry, College of Pharmacy, Gachon University, Incheon, Republic of Korea; Federico II University of Naples, ITALY

## Abstract

Paradols are non-pungent and biotransformed metabolites of shogaols and reduce inflammatory responses as well as oxidative stress as shogaols. Recently, shogaol has been noted to possess therapeutic potential against several central nervous system (CNS) disorders, including cerebral ischemia, by reducing neuroinflammation in microglia. Therefore, paradol could be used to improve neuroinflammation-associated CNS disorders. Here, we synthesized paradol derivatives (2- to 10-paradols). Through the initial screening for anti-inflammatory activities using lipopolysaccharide (LPS)-stimulated BV2 microglia, 6-paradol was chosen to be the most effective compound without cytotoxicity. Pretreatment with 6-paradol reduced neuroinflammatory responses in LPS-stimulated BV2 microglia by a concentration-dependent manner, which includes reduced NO production by inhibiting iNOS upregulation and lowered secretion of proinflammatory cytokines (IL-6 and TNF-α). To pursue whether the beneficial *in vitro* effects of 6-paradol leads towards *in vivo* therapeutic effects on transient focal cerebral ischemia characterized by neuroinflammation, we employed middle cerebral artery occlusion (MCAO)/reperfusion (M/R). Administration of 6-paradol immediately after reperfusion significantly reduced brain damage in M/R-challenged mice as assessed by brain infarction, neurological deficit, and neural cell survival and death. Furthermore, as observed in cultured microglia, 6-paradol administration markedly reduced neuroinflammation in M/R-challenged brains by attenuating microglial activation and reducing the number of cells expressing iNOS and TNF-α, both of which are known to be produced in microglia following M/R challenge. Collectively, this study provides evidences that 6-paradol effectively protects brain after cerebral ischemia, likely by attenuating neuroinflammation in microglia, suggesting it as a potential therapeutic agent to treat cerebral ischemia.

## Introduction

Nutraceuticals derived from spices such as turmeric, ginger, and garlic have been demonstrated to regulate central nervous system (CNS) disorders by modulating inflammatory pathways. Numerous lines of evidence indicate that spice–derived nutraceuticals may prevent neurodegenerative diseases. Interestingly, epidemiological data reveals that populations in places like the Indian subcontinent, where people regularly consume spices, have a lower prevalence of neurodegenerative diseases compared with those of countries in the western world [1]. This includes spices such as turmeric, red pepper, black pepper, licorice, clove, ginger, garlic, coriander, and cinnamon. [2,3,4,5]. Several reports have emphasized ginger as a beneficial nutraceutical, particularly for CNS disorders [6,7,8]. Ginger oils are a series of natural components from *Zinginber officinale* and they are classified according to their alkyl chain length, e.g. 4-, 6-, or 8-gingerol. Many previous studies have reported that ginger oils exert potential effects against cancer, skin problems, gastrointestinal tract diseases, and CNS disorders associated with oxidative and inflammatory stresses [9]. Gingerols, gingerone, shogaol, and paradol are main functional ingredients of ginger oils [10]. Interestingly, the dehydrated form of 6-gingerol, 6-shogaol, is more active [11,12,13,14]. The pharmacological properties of 6-shogaol have been reported to be beneficial in a wide variety of CNS disorders, such as Parkinson’s disease (PD), Alzheimer’s disease (AD), sepsis-induced neuroinflammation, and cerebral ischemia [12,15,16]. The main properties of 6-shogaol’s protective effects in these disorders are closely associated with its anti-inflammatory and anti-oxidative properties. In our previous study, 6-shogaol was revealed to be neuroprotective in the septic brain or transient global ischemia via the attenuation of microglial activation [12], a key component of neuroinflammation that is a feature in many CNS disorders [17,18].

Recently, non-pungent and relatively stable paradol has been identified as a metabolite of shogaol by liver enzymatic reduction. Paradol also possesses anti-inflammatory and anti-oxidative activities as shogaol does [19,20,21,22]. Because of this, paradol derivatives may have attracted attention as a potential candidate for drug discovery to cure neuroinflammation-associated CNS disorders, particularly in cerebral ischemia. However, there is no clear report that deals with the neuroprotective effect of paradol in these CNS disorders. Therefore, in the current study, we primarily synthesized five paradol derivatives, such as 2-, 4-, 6-, 8-, and 10-paradol, and selected 6-paradol as the most effective compound with anti-inflammatory effect in lipopolysaccharide (LPS)-stimulated BV2 microglia. Furthermore, we have assessed the neuroprotective effect of synthetic 6-paradol by evaluating its anti-neuroinflammatory effect *in vitro* and *in vivo* using cultured microglia and a mouse model of transient focal cerebral ischemia.

## Materials and Methods

### General procedure of reduction of shogaols to paradols

Palladium on charcoal (10 mol%) was added to a solution of α,β-unsaturated ketone **5** (shogaol) (0.1 ~1.0 mmol scale) in methanol (0.05 M). The reaction mixture was stirred under H_2_ gas (balloon) atmosphere for about 30 minute at room temperature. After checking for the complete disappearance of starting materials with thin layer chromatography, the mixture was filtered using a short celite pad and the filtrate was concentrated under reduced pressure. The residue was purified by SiO_2_ column chromatography to give the desired product.

#### 2-Paradol [1-(4-hydroxy-3-methoxyphenyl)hexan-3-one] (6a)

SiO_2_ column chromatography (ethyl acetate: hexane = 1:7); yield: 94%; ^1^H-NMR (600MHz, CDCl_3_) δ (ppm): 6.82 (d, *J* = 8.4 Hz, 1H), 6.69–6.65 (m, 2H), 5.4 7 (s, 1H), 3.87 (s, 3H), 2.82 (dd, *J* = 7.2Hz, 7.8Hz, 2H), 2.69 (dd, *J* = 7,2Hz 7.8Hz, 2H), 2.36 (dd, *J* = 7.2Hz, 7.8Hz, 2H), 1.62–1.55 (m, 4H), 0.89 (t, *J* = 7.8Hz, 3H); ^13^C-NMR (150MHz, CDCl_3_) δ (ppm) 210.5, 146.4, 143.9, 133.1, 120.8, 114.3, 111.0, 55.9, 45.0, 44.7, 29.5, 17.2, 13.8

#### 4-Paradol [1-(4-hydroxy-3-methoxyphenyl)octan-3-one] (6b)

SiO_2_ column chromatography (ethyl acetate: hexane = 1:8); yield = 95%; ^1^H-NMR (600MHz, CDCl_3_) δ (ppm): 6.82 (d, *J* = 8.4Hz,1H), 6.69–6.65 (m, 2H), 5.46 (s,1H), 3.87 (s, 3H), 2.82 (t, *J* = 7.2Hz, 7.8Hz, 2H), 2.69 (t, *J* = 7.8Hz, 2H), 2.36 (t, *J* = 7.8Hz, 7.2Hz, 2H) 1.58–1.52 (m, 2H), 1.32–1.20(m, 4H), 0.87 (dd, *J* = 6.6Hz, 7.2 Hz, 3H); ^13^C-NMR (150MHz, CDCl_3_) δ (ppm) 210.6, 146.4, 143.9, 133.1, 120.8, 114.3, 111.0, 55.9, 44.6, 43.1, 31.4, 29.5, 23.5, 22.4, 13.9

#### 6-Paradol [1-(4-hydroxy-3-methoxyphenyl)decan-3-one] (6c)

SiO_2_ column chromatography (ethyl acetate: hexane = 1:8); yield = 90%; ^1^H-NMR (600MHz, CDCl_3_) δ(ppm) 6.82 (d, *J* = 7.8Hz,1H), 6.69–6.65 (m, 2H), 5.46 (s,1H), 3.87 (s, 3H), 2.82 (t, *J* = 7.2Hz, 7.8Hz, 2H), 2.69 (t, *J* = 7.8Hz, 7.2Hz, 2H), 2.36 (t, *J* = 7.8Hz, 7.2Hz, 2H) 1.57–1.52 (m, 2H), 1.29–1.21 (m, 8H), 0.87 (t, *J* = 6.6Hz, 7.2 Hz, 3H); ^13^C-NMR (150MHz, CDCl_3_) δ (ppm) 210.6, 146.4, 143.9, 133.1, 120.8, 114.3, 111.0, 55.9, 44.6, 43.2, 31.7, 29.5, 29.2, 29.1, 23.8, 22.6, 14.1

#### 8-Paradol [1-(4-hydroxy-3-methoxyphenyl)dodecan-3-one] (6d)

SiO_2_ column chromatography (ethyl acetate: hexane = 1:8); yield = 90%; ^1^H-NMR (600MHz, CDCl_3_) δ (ppm): 6.82 (d, *J* = 8.4Hz,1H), 6.69–6.65 (m, 2H), 5.46 (s,1H), 3.87 (s, 3H), 2.82 (t, *J* = 7.2Hz, 7.8Hz, 2H), 2.69 (t, *J* = 7.8Hz, 7.2Hz, 2H), 2.36 (t, *J* = 7.2Hz, 2H) 1.56–1.53 (m, 2H), 1.30–1.24 (m, 12H), 0.87 (t, *J* = 7.2 Hz, 6.6Hz, 3H); ^13^C-NMR (150MHz, CDCl_3_) δ (ppm) 210.6, 146.4, 143.8, 133.1, 120.8, 114.3, 111.0, 55.9, 44.6, 43.2, 31.9, 29.5, 29.4, 29.4, 29.3, 29. 2, 23.8, 22.7, 14.1

#### 10-Paradol [1-(4-hydroxy-3-methoxyphenyl)tetradecan-3-one] (6e)

SiO_2_ column chromatography (ethyl acetate: hexane = 1:9); yield = 87%; ^1^H-NMR (600MHz, CDCl_3_) δ (ppm) 6.82 (d, *J* = 8.4Hz,1H), 6.69–6.65 (m, 2H), 5.50 (s,1H), 3.86 (s, 3H), 2.82 (t, *J* = 7.2Hz, 7.8Hz, 2H), 2.69 (t, *J* = 7.8Hz, 7.2Hz, 2H), 2.36 (t, *J* = 7.2Hz, 7.8Hz, 2H) 1.56–1.53 (m, 2H), 1.30–1.24 (m, 16H), 0.87 (t, *J* = 7.2 Hz, 3H); ^13^C-NMR (150MHz, CDCl_3_) δ (ppm): 210.6, 146.4, 143.9, 133.2, 120.8, 114.3, 111.1, 55.9, 44.6, 43.2, 31.9, 29.6, 29.4, 29.5, 29.4, 29.3, 29.2, 23.8, 22.7, 14.1.

### BV-2 microglia culture and treatment

Murine microglial BV-2 cells, generously provided by Dr. E. Choi (Korea University, Seoul, Korea), were cultivated, as described previously [12]. BV-2 cells were then plated on the appropriate culture plates and pretreated with synthesized paradols for 30 min followed by an exposure to LPS (100 ng/ml) for 24 h.

### Determination of nitric oxide (NO) productivity and cell viability (MTT assay)

BV-2 cells grown on 96-well plates (3×10^4^ cells/well) were used for these assays. After treatment, the concentration of nitrite (NO_2_) that accumulated in the 50 μL conditioned medium (CM) was determined using the Griess reaction and cell viability was measured after the cells were incubated with 3-[4,5-dimethylthiazol-2-yl]-2,5-diphenyl-tetrazolium bromide (MTT), as described previously [12].

### Measurement of interleukin-6 (IL-6) and tumor necrosis factor-α (TNF-α)

In the CM obtained from cells grown on 24-well plates (3×10^5^ cells/well), concentrations of TNF-α and IL-6 were measured by competitive enzyme immunoassay kits (R&D Systems, Minneapolis, MN, USA) according to the manufacture’s protocol.

### Western blot analysis

Proteins obtained from BV-2 cells (6×10^5^ cells/well in a 6-well plate) were used for Western blot analysis. Total proteins (30 μg) of each group were separated by 10% SDS-PAGE gel electrophoresis, transferred to a nitrocellulose membranes, and incubated with primary antibodies (Cell Signaling, Beverly, MA, USA) against α-tubulin or iNOS. Membranes were incubated with horseradish peroxidase (HRP)-conjugated secondary antibodies (Cell Signaling), and protein bands were visualized using an ECL Western Blotting Detection Reagents (Amersham Pharmacia Biotech, Buckinghamshire, UK). Densitometry analysis of the bands was performed using ImageMaster 2D Elite software (version 3.1, Amersham, Pharmacia Biotech).

### Quantitative real-time PCR

Total RNA (1 μg) was isolated from the ipsilateral cortex and striatum of each group. Synthesized cDNA by a reverse transcription was used for quantitative real-time PCR (qRT-PCR). Targets including iNOS and TNF-α were amplified with Brilliant III Ultra-Fast SYBR^®^ Green mix (Agilent) on an M×3005p system (Stratagene, La Jolla, USA) using gene-specific primer pairs (S1 Table).

### Induction of transient middle cerebral artery occlusion (MCAO)/reperfusion (M/R) in mice

All mouse handling procedures were performed in accordance with approved animal protocols by the Institutional Animal Care and Use Committee at Gachon University (Incheon, Republic of Korea) (# of approved animal protocols: LCDI-2012–0075 and LCDI-2013–0074). Male ICR mice (7 weeks old, 36 ± 2 g; Orient Co., Ltd. (Korea), a branch of Charles River Laboratories) were housed 3 or 4 per cage under the controlled condition of 12 h light/dark cycle, temperature (24 ± 2°C), and a relative humidity (60 ± 10%), with free access to food and water. After a week of laboratory acclimatization, mice were challenged with M/R as described previously [23]. Briefly, mice were ventrally fixed in an operating frame with the temperature set as 37°C and anesthetized with isoflurane (3% for induction and 1.5% for maintenance) in N_2_O∶O_2_ (3∶1). A ventral neck incision was made and the right common carotid artery (CCA) was exposed and carefully separated from the vagus nerve. MCAO was induced by inserting a 9-mm-long 5–0 nylon monofilament coated with silicon from the CCA bifurcation to the MCA. Blood flow was restored 90 min after MCAO by withdrawing the monofilament. The same surgical procedure, except for the occlusion, was carried out for sham group. After M/R surgery, mice were housed 3 per cage with moist food and soft bedding materials to reduce suffering until they were sacrificed by CO_2_ inhalation or used for sampling.

### Administration of 6-paradol

Mice challenged with M/R were randomly divided into vehicle (10% Tween80)- or 6-paradol-administered groups (n = 6~7 per group). 6-Paradol dissolved in 10% Tween80 was orally administered into mice at 1, 5, or 10 mg/kg immediately after reperfusion.

### Determination of infarct volume and functional neurological deficit score

Modified neurological severity score (mNSS) was used to assess motor function, sensory function, reflex, and balance 22 h after reperfusion. The sum of partial scores yielded the total mNSS with a maximum of 18 points and minimum of 0 in normal mice as previously described [24].

After obtaining the neurological score, brains were quickly removed after CO_2_ exposure and sectioned into 2 mm thick coronal sections. Brain slices were then stained using 2% 2,3,5-triphenyltetrazolium chloride (TTC) in physiological saline at 37°C. TTC stained slices were photographed and infarct volume was analyzed by dividing the infarct portion through the total volume of the slices using ImageJ software (National Institute of Mental Health, Bethesda, MD).

### Histology

In general, samples for histological analysis were obtained 22 h after reperfusion. Alternatively, brain samples were obtained 3 days after reperfusion to examine morphological response and proliferation of microglia. Anesthetized mice with a combination of Zoletil 50^®^ (10 mg/kg, i.m.) and Rompun^®^ (3 mg/kg, i.m.) were perfused with PBS (pH 7.4) followed by ice-cold 4% paraformaldehyde. Removed brains were incubated in fixative and 30% sucrose solution, embedded in Tissue-Tek Optimal Cutting Temperature (OCT) compound, frozen on dry ice, and cut into 20 μm sections on cryostat (J4800AMNZ, Thermo, Germany). Bright field or fluorescent images were collected with a microscope (BX53T, Olympus, Japan) equipped with a DP72 camera or laser scanning confocal microscopy (Eclipse A1 Plus, Nikon, Japan) and representative images were prepared by Adobe Photoshop CS3.

#### Nissl staining

Brain sections were treated overnight with blocking solution and chloroform/ethanol (1:1), followed by rehydration (100% and 95% ethanol solution for 5 min at each step) and washed with the dH_2_O for 5 min. Sections were stained with 0.5% cresyl violet acetate, dehydrated with 95% ethanol for 5 min, 100% ethanol for 10 min, and Xylene for 10 min, and mounted using mounting media.

#### Fluoro-Jade B staining

Brain sections were rinsed with the dH_2_O, rehydrated (100% ethanol for 3 min, 70% ethanol for 1 min, and 30% ethanol for 1 min), and washed with dH_2_O for 1 min. Sections were soaked in 0.06% potassium permanganate for 15 min for oxidation, washed with dH_2_O, and incubated with 0.001% solution of Fluoro-Jade B dissolved in 0.09% acetic acid for 30 min. They were rinsed three times with dH_2_O, dried on the slide warmer, dehydrated with xylene, and mounted with the mounting media.

#### Immunohistochemistry

Brain sections were treated with 1% hydrogen peroxide in PBS for 15 min and incubated for 1 h with 5% normal donkey serum in PBS containing 0.5% Triton X-100 to block nonspecific protein binding. Sections were labeled with primary antibodies against Iba1 (1:500, Abcam), GFAP (1:500, Sigma), iNOS (1:500, Abcam), or TNF-α (1:100, Abcam), followed by labeling with the appropriate secondary biotinylated antibodies (1:200) and avidin/biotin complex (ABC, 1:100, Vector Labs). Signals were visualized with DAB staining (0.02% DAB and 0.01% H_2_O_2_ for 5 min).

#### Bromodeoxyuridine (BrdU) immunofluorescence

To examine microglial proliferation after M/R challenge, BrdU (50 mg/kg, i.p.; Sigma-Aldrich) was injected twice daily at 2-h interval for 2 days (1 and 2 days after M/R challenge), as described previously [25]. Brain samples were obtained 3 days after M/R challenge from mice perfused under anesthesia using a combination of Zoletil^®^ and Rompun^®^ and tissue sections (20 μm) were processed for double immunofluorescence against BrdU and Iba1 to determine microglial proliferation, as described previously [26]. Sections were incubated with HCl (2 N) at 37°C and neutralized with borate buffer (0.1 M, pH 8.5) for 3 × 15 min. Sections were blocked with 1% FBS and labeled overnight at 4°C with primary antibodies against BrdU (1:200, Abcam) and Iba1 followed by labeling with secondary antibodies conjugated with Cy3 (1: 1000; Jackson ImmunoResearch) and AF488 (1: 1000; Invitrogen), respectively. Fluorescent images were captured with laser scanning confocal microscopy.

### Statistical Analysis

The data were analyzed using Statistical Analysis System (SAS) software (PRISM). All data are expressed as mean ± S.E.M. Differences among the groups were analyzed by a one-way ANOVA followed by *Newman-Kleus* test or *Dunnett’s* test for multiple comparison. P value<0.05 was considered as statistically significant for the experimental analysis.

## Results

### Synthesis of paradols

**Fig 1:**
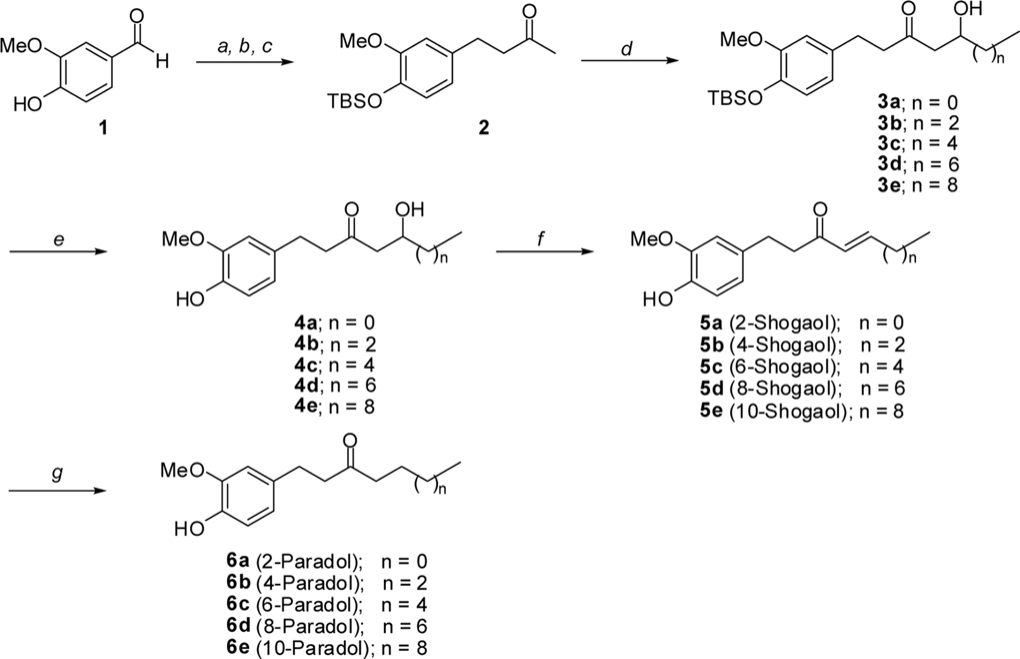
Syntheses of 2-, 4-, 6-, 8-, and 10-paradol. Reagents and Conditions; a. TBSCl, imidazole, CH_2_Cl_2_, rt, 1 h, 93%. b. acetone, NaOH, H_2_O, rt, 6 h, 84% c. Pd/C, H_2_, MeOH, rt, 1 h, 86% d. LDA, THF, -78°C; then acetaldehyde, n-butanal, n-hexanal, n-octanal, or n-decanal, 60 ~ 80% e. TBAF, THF, 1 h, 84 ~ 92% f. *p*-TsOH, benzene, rt, 2 day, 65 ~ 82% g. Pd/C, H2, MeOH, rt, 0.5 h, 87 ~ 95%.

Syntheses of 2-, 4-, 6-, 8-, and 10-paradol were shown in Fig. 1. Shogaols (5a–5f), synthetic precursors of paradols, were prepared using a modified procedure for shogaol synthesis. Vanillin (1) was transformed to ketone 2 through three sequential steps, with the phenolic hydroxyl protected with t-butyldimethylsilyl group, by an aldol reaction with acetone, and olefin reduction by catalytic hydrogenation with palladium on charcoal. Selective abstraction of terminal proton of ketone by treatment of lithium diisopropylamide at -78°C generated less substituted enolate, and then, addition of the corresponding aldehydes to give β-hydroxyketones (3a–3f). In this study, five aldehydes, acetaldehyde, n-butanal, n-hexanal, n-octanal, and n-decanal were used. Removal of *tert*-Butyldimethylsilyl (TBS) group by *tetra*-n-butylammonium fluoride (TBAF) produced gingerols (4a–4f), followed by dehydration to yield the shogaols series (5a–5f). Finally, olefine in shogaols was saturated by hydrogenation to create the desired 2-, 4-, 6-, 8-, and 10-paradols (6a–6f) in high yields.

### 6-Paradol reduces inflammatory responses of activated BV2 microglia

The goal of this study is to provide evidences that paradol, a non-pungent metabolite of shogaol, exerts its neuroprotective effects via anti-inflammatory activities as shogaol does. For this purpose, we have determined production of NO and proinflammatory cytokines, all of which were reported to be blocked by shogaol in LPS-stimulated BV2 microglia [12].

To determine, at first, which synthesized paradol derivative effectively reduced neuroinflammation, we assessed NO production and MTT reduction of LPS-stimulated BV2 microglia that were pretreated with 5 of the paradol derivatives (2-, 4-, 6-, 8-, or 10-paradol) at 10 μg/mL prior to LPS exposure. All species of paradol reduced LPS-induced NO production (S1A Fig.). Among the tested compounds, 2- or 6-paradol increased cell viability whereas 8- or 10-paradol caused cytotoxicity in LPS-stimulated BV2 cells (S1B Fig.). 6-Shogaol (10 μg/mL) was used as a positive control and reduced NO production to the same extent as 6-paradol (S1A Fig.). 6-Shogaol did not affect cell viability compared with LPS alone, but which was slightly different than 6-paradol’s effects (S1B Fig.). Based on these data, 6-paradol appeared to be the most effective paradol derivative. Therefore, we have used 6-paradol throughout this study.

**Fig 2:**
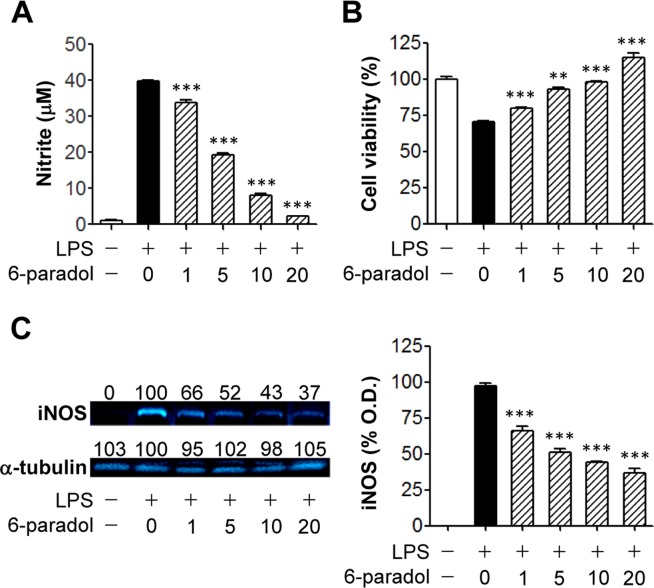
Pretreatment with 6-paradol reduces NO production, cytotoxicity, and iNOS upregulation in LPS-stimulated BV2 microglia. Cells were pretreated with different concentrations of 6-paradol (0, 1, 5, 10, or 20 μg/ml) for 30 min and stimulated with 100 ng/ml LPS for 24 h. (A) The amount of nitrite accumulated in conditioned medium was measured using ELISA. (B) Cell viability was assessed using MTT assay. n = 3 per group. **p<0.01 and ***p<0.001, versus LPS alone. (C) Expression of iNOS protein was determined by Western blot in cell lysates. Representative Western blots (*left*). Optical density of iNOS-specific bands (*right*). Values presented per each band indicate fold decrease compared with LPS alone. n = 3 per group. ***p<0.001, versus LPS alone.

When 6-paradol (1 to 20 μg/mL) was added to cultures of BV2 microglia after they were exposed to LPS for 24 h, 6-paradol reduced NO production (Fig. 2A) and increased cell viability (MTT assay, Fig. 2B) in a concentration-dependent manner. In addition, LPS-stimulated BV2 cells underwent apoptosis even with a very small population, which was significantly attenuated by 20 μM 6-paradol (S2 Fig.). The reduced NO production by 6-paradol was mediated by the attenuation of LPS-induced iNOS upregulation (Fig. 2C, D).

**Fig 3:**
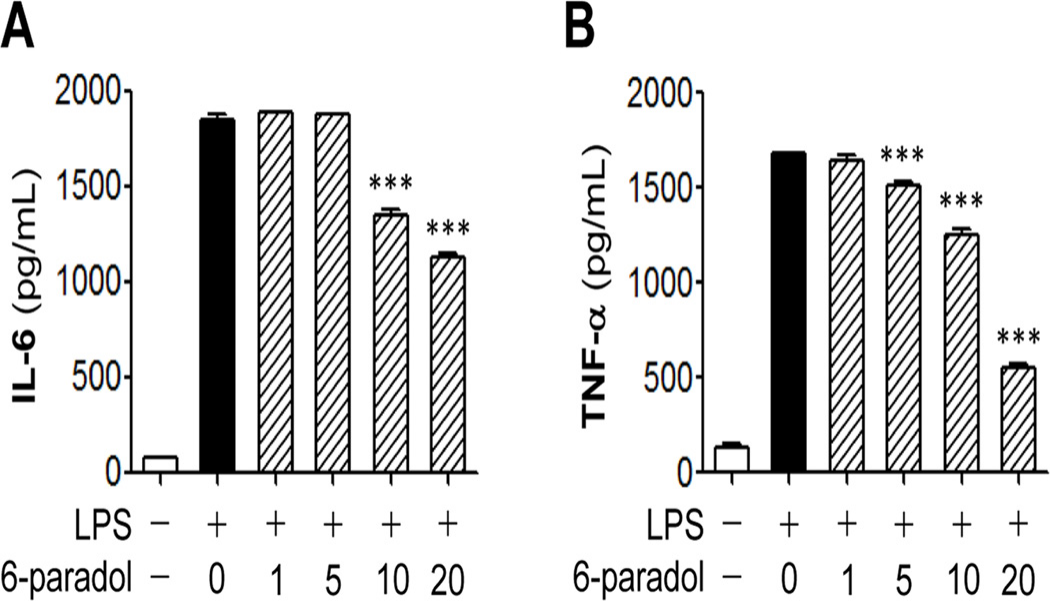
Pretreatment with 6-paradol reduces secretion of IL-6 and TNF-α from LPA- stimulated BV2 microglia. Cells were pretreated with different concentrations of 6-paradol (0, 1, 5, 10, or 20 μg/ml) for 30 min and stimulated with 100 ng/ml LPS for 24 h. Levels of IL-6 or TNF-α were determined in conditioned medium using an enzyme-based immunoassay kit. n = 3 per group. ***p<0.001, versus LPS alone.

To assess whether 6-paradol effectively reduced the secretion of proinflammatory cytokines in stimulated microglia, we measured IL-6 (Fig. 3A) and TNF-α (Fig. 3B) production by enzyme immunoassay. We observed that 6-paradol blocked the secretion of both cytokines in a concentration-dependent manner, with a more potent effect on TNF-α production (Fig. 3).

### 6-Paradol reduces brain damages induced by transient focal cerebral ischemia

To evaluate whether *in vitro* anti-inflammatory activities of 6-paradol are linked into a therapeutic effect *in vivo*, 6-paradol was tested in a mouse model of transient focal cerebral ischemia where neuroinflammation is a main pathogenetic event. Mice subjected to MCAO (90 min) were challenged with 6-paradol (1, 5, or 10 mg/kg; p.o.) immediately after reperfusion. Brain damage was assessed 22 h after reperfusion, which includes brain infarction, neurological deficit, neural cell survival/death, and neuroinflammatory markers-associated with the *in vitro* effects of 6-paradol.

**Fig 4:**
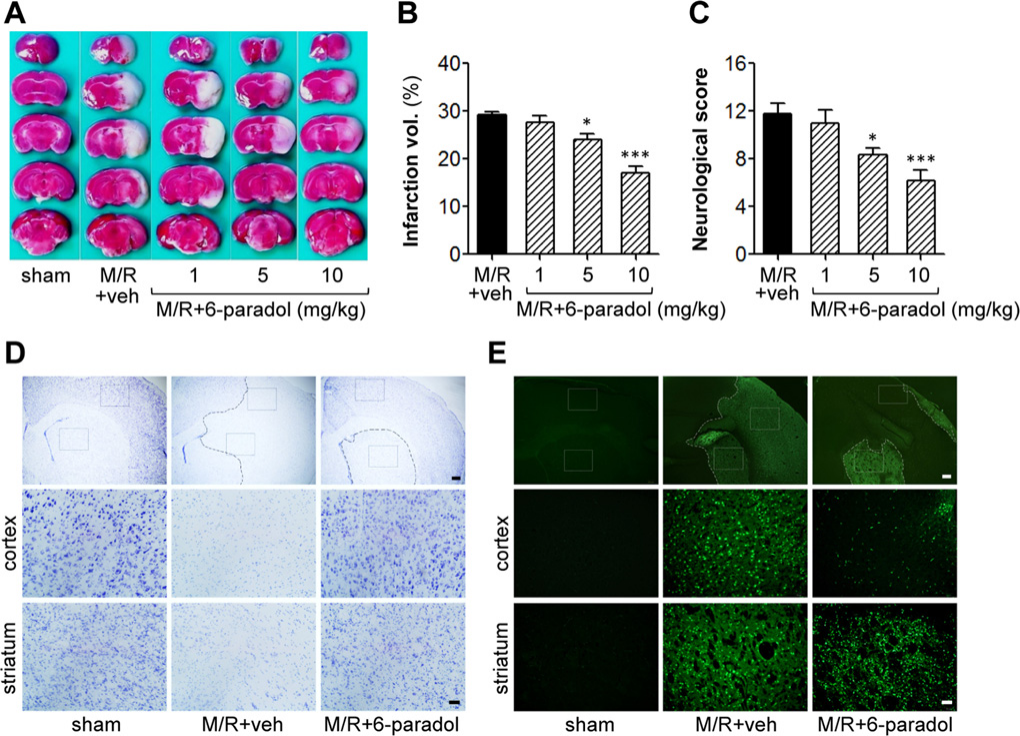
Administration of 6-paradol reduces brain damage in M/R-challenged mice. Mice were challenged with M/R and 6-paradol (1, 5, or 10 mg/kg, p.o.) was administered immediately after reperfusion. Brain damage was assessed 22 h after reperfusion. (A-C) Effects of 6-paradol at different dosages (1 to 10 mg/kg) on infarct volume (A, B) and neurological function (C) were determined. Representative images of TTC-stained brain tissue (A) and quantification of brain infarction (B). Neurological score indicating neurological functions (C). n = 6~7 per group. *p<0.05 and ***p<0.001, versus vehicle-administered M/R mice (M/R+veh). (D, E) Effects of 6-paradol (10 mg/kg) on neural cell survival (D) and death (E) were determined by staining with cresyl violet (Nissl staining) and Fluoro-Jade B, respectively. In both cases, representative images were shown. Dashed lines indicate the lesion site. Diagram boxes display the cerebral area where the images in middle and bottom panels were acquired. Scale bars, 200 μm (top panels) and 50 μm (middle and bottom panels) in D and E.

Firstly, the therapeutic potential of 6-paradol on cerebral ischemia was assessed. Oral administration of 6-paradol reduced brain infarction (Fig. 4A, B) and improved the neurological score (Fig. 4C) in a dose-dependent manner. At 10 mg/kg, 6-paradol remarkably reduced brain infarction and improved neurological score by 42.1% (Fig. 4B) and 49.2% (Fig. 4C), respectively, compared to the vehicle-treated M/R group. These neuroprotective effects of 6-paradol (10 mg/kg) were confirmed by determining cell survival or death by staining with Nissl (Fig. 4D) or Fluoro-Jade B (Fig. 4E). In a 6-paradol-administered group, the survival of neural cells was higher than the vehicle-treated group. Similarly, neural cell death was significantly reduced in the 6-paradol group. The observed neuroprotection by 6-paradol administration was similar to that of 6-shogaol (10 mg/kg, p.o.) when the effects were compared at the same dosage (S3 Fig.).

**Fig 5:**
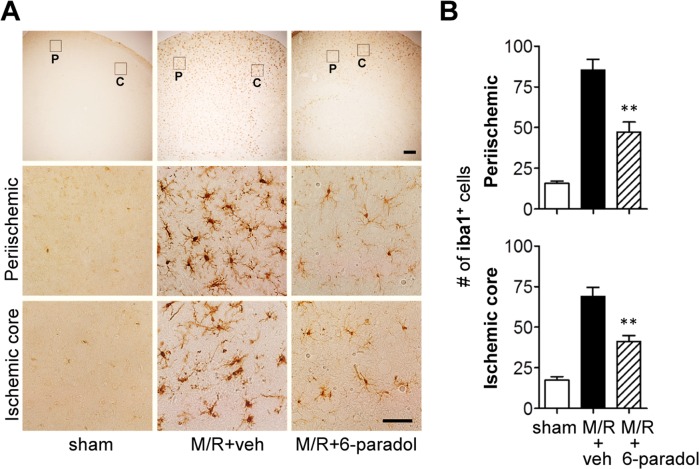
Administration of 6-paradol reduces microglial activation in the post-ischemic brain 1 day after M/R challenge. Mice were challenged with M/R and 6-paradol (10 mg/kg, p.o.) was administered immediately after reperfusion. Microglial activation was assessed 22 h after reperfusion by immunohistochemistry against Iba1. (A) Representative images of Iba1-immunopositive cells in periischemic (‘P’) and ischemic core (‘C’) regions. Scale bars, 200 μm (top panels) and 50 μm (middle and bottom panels). (B) Quantification of Iba1-immunopositive cells in both regions. n = 4~5 per group. **p<0.01, versus vehicle-administered M/R mice (M/R+veh).

**Fig 6:**
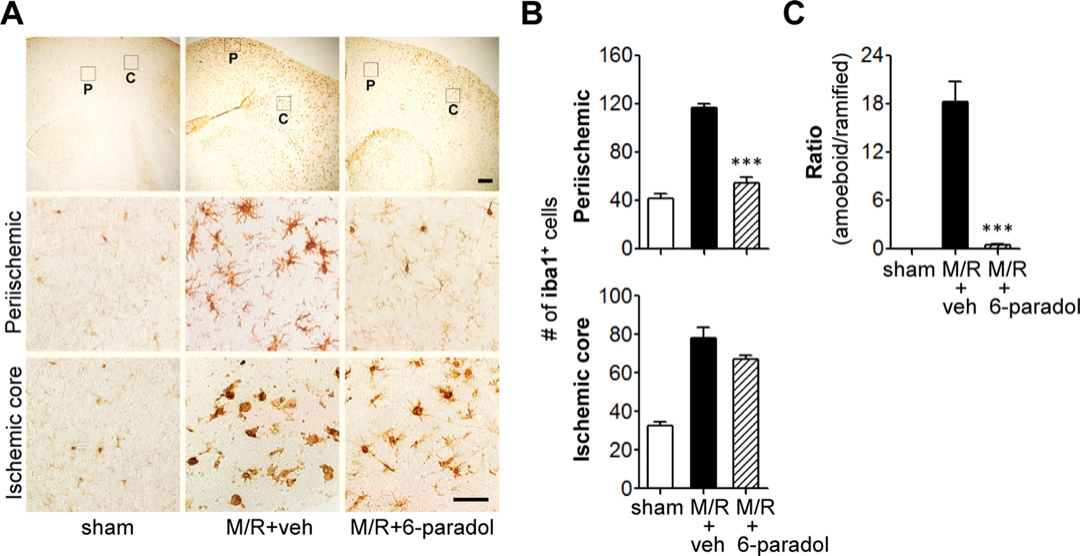
Administration of 6-paradol reduces microglial activation in the post-ischemic brain 3 days after M/R challenge. Mice were challenged with M/R and 6-paradol (10 mg/kg, p.o.) was administered immediately after reperfusion. Microglial activation was assessed 3 days after reperfusion by immunohistochemistry using Iba1 antibody. (A) Representative images of Iba1-immunopositive cells in periischemic (‘P’) and ischemic core (‘C’) regions. Scale bars, 200 μm (top panels) and 50 μm (middle and bottom panels). (B) Quantification of Iba1-immunopositive cells in both regions. n = 6~7 per group. (C) Quantification of morphological changes of Iba1-positive cells in ischemic core regions (from ‘ramified’ into ‘amoeboid’ cells). ***p<0.001, versus vehicle-administered M/R mice (M/R+veh).

**Fig 7:**
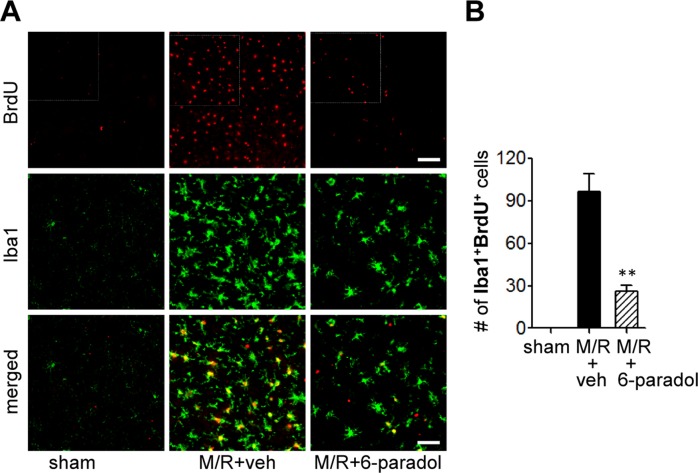
Administration of 6-paradol reduces microglial proliferation in the post-ischemic brain 3 days after M/R challenge. Mice were challenged with M/R and 6-paradol (10 mg/kg, p.o.) was administered immediately after reperfusion. Microglial proliferation was assessed 3 days after reperfusion by double immunolabeling using antibodies against Iba1 and BrdU. (A) Representative images of Iba1-immunopositive cells in the marginal zone (zone between periischemic and core regions). Scale bars, 100 μm (top panels) and 50 μm (middle and bottom panels). Diagram boxes display the cerebral area where the images were acquired. Low magnification images for BrdU labeling from an independent experiment were shown as S4 Fig. (B) Quantification of Iba1/BrdU-double-positive cells. n = 3~4 per group. **p<0.01, versus vehicle-administered M/R mice (M/R+veh).

To determine whether the neuroprotective effect of 6-paradol is associated with its anti-inflammatory activities *in vitro*, we assessed microglial activation using immunohistochemical analyses. At first, we examined effects of 6-paradol on well-characterized microglial responses in the different regions 1 and 3 days after M/R as assessed by the increased number of Iba1-immunopositive cells [27,28]. As reported, the number of Iba1-postive cells was markedly increased in both periischemic and ischemic core regions 1 (Fig. 5) and 3 days (Fig. 6A, B) after M/R challenge. Morphological changes of Iba1-positive cells were also obvious in core regions 3 days after M/R challenge (ramified → amoeboid) (Fig. 6A, C). Administration of 6-paradol (10 mg/kg) clearly reduced the number of Iba1-positive cells 1 and 3 days after the challenge (Fig. 5 and Fig. 6A, B). Moreover, 6-paradol dramatically reduced the number of Iba1-postive cells in periischemic regions even after 3 days following M/R challenge (Fig. 6A, B). In ischemic core regions, 6-paradol seemed not to reduce the number of Iba1-positive cells at 3 days following M/R challenge (Fig. 6A, B). But, interestingly, 6-paradol dramatically reversed microglial morphology into ‘ramified’ in ischemic core regions (Fig. 6A, C) despite of no effect on the number of cells bearing Iba1 (Fig. 6A, B). These effects of 6-paradol on microglial responses were further examined by assessing microglial proliferation (Fig. 7 and S4 Fig.). The number of BrdU-positive cells was markedly increased in brains 3 days after M/R and most of BrdU-positive cells also expressed Iba1 (Fig. 7 and S4 Fig.), demonstrating microglial proliferation in the post-ischemic brains. Administration of 6-paradol significantly reduced the number of Iba1/BrdU-double-positive cells (Fig. 7 and S4 Fig.). These data demonstrated that 6-paradol effectively attenuated microglial responses in the post-ischemic brains.

**Fig 8:**
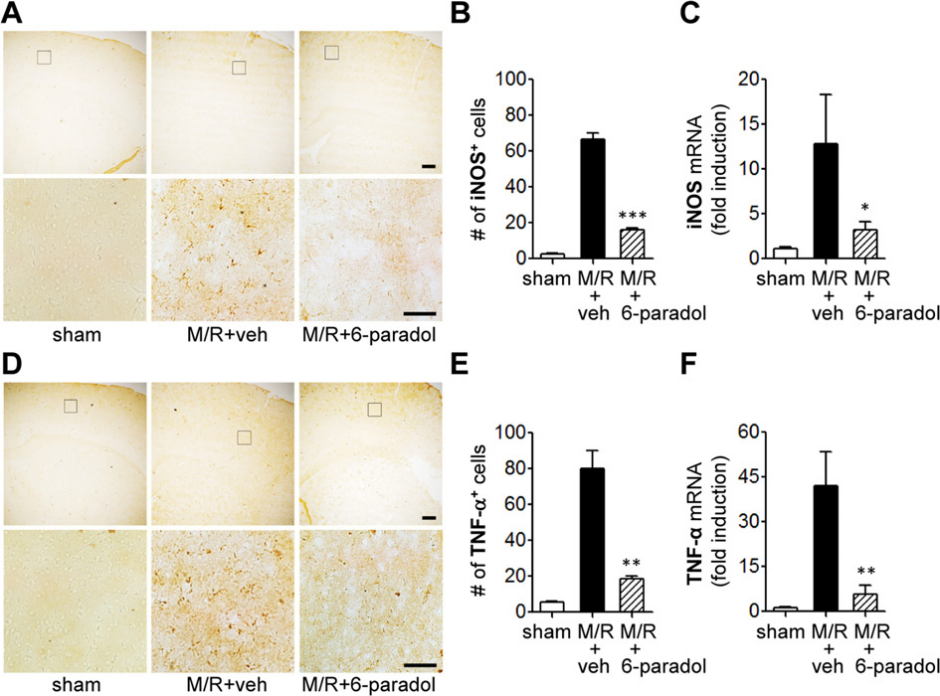
Administration of 6-paradol reduces expression of iNOS and TNF-α in M/R-challenged brain. Mice were challenged with M/R and 6-paradol (10 mg/kg, p.o.) was administered immediately after reperfusion. Expression of iNOS and TNF-α proteins and mRNA was assessed 22 h after reperfusion by immunohistochemistry and qRT-PCR analysis, respectively. (A-C) Representative images (A) and quantification (B) of iNOS-immunopositive cells. n = 4 per group. (C) Changes in expression of iNOS mRNA in the ipsilateral cortex. n = 4~5 per group. (D-F) Representative images (D) and quantification (E) of TNF-α-immunopositive cells. n = 4 per group. (F) Changes in expression of TNF-α mRNA in the ipsilateral cortex. n = 4~5 per group. *p<0.05, **p<0.01, and ***p<0.001, versus vehicle-administered M/R mice (M/R+veh). Scale bars, 200 μm (upper panels) and 50 μm (lower panels) in A and D. Diagram boxes in A and D display the cerebral area where the images were acquired.

We also determined whether *in vitro* anti-inflammatory effects of 6-paradol on iNOS and TNF-α proteins were reaffirmed in the post-ischemic brains using immunohistochemical and qRT-PCR analyses. Upregulation of iNOS (Fig. 8A-C) and TNF-α (Fig. 8D-F) in M/R-challenged brains was also reversed by 6-paradol administration (Fig. 8). Upon M/R challenge, the upregulation was evident in cortex regions (Fig. 8), but not in striatum regions where we could not detect any positive signals (data not shown).

## Discussion

The aggregate *in vitro* and *in vivo* results demonstrate that 6-paradol, a non-pungent metabolite of 6-shogaol, is a novel active component of *Zinginber officinale* with a therapeutic potential on transient focal cerebral ischemia possibly via an inhibition of neuroinflammatory responses in activated microglia. In activated microglia, a robust increase in NO production and proinflammatory cytokines (i.e., IL-6 and TNF-α) was markedly blocked by exposure to 6-paradol, indicating that it may function as a neuroprotectant that reduces inflammatory responses. These *in vitro* neuroprotective effects were reaffirmed in an animal model of cerebral ischemia where 6-paradol showed therapeutic benefits by reducing microglial activation and TNF-α expression.

Paradols, olefin-reduced form of shogaols, are the major compounds of thermally processed Ginger extract. They are also found in nature and thought to be biological metabolites of shogaols. Recently, there have been a few reports where the chain lengths of shogaols or paradols affect their pharmacokinetics and biological activities, such as neuronal protection from β-amyloid formation and antiobesity activity [29,30,31]. One study reported that shogaols with longer chain (4- to 12-shogarol) had better neuroprotection [30]. Many researchers who have extensively studied 6-shogaol have demonstrated that it possesses several biologically important activities against cancer, neuronal damage, and inflammation [12,15,16,32,33], which can support our results. In this study, it appears that 6-paradol is the most effective one among the tested paradol derivatives in reducing inflammatory responses in activated microglia. In fact, our notion can be further supported by several reports where 6-paradol is the most effective compound for modulating obesity, platelet aggregation, or 12-*O*-tetradecanoylphorbol-13-acetate-induced alterations when compared to other paradol derivatives [19,29,34].

Brain damage in cerebral ischemia is triggered by diverse pathogenetic events occurring in diverse cell types, including energy failure, excitotoxicity, oxidative stress, and neuroinflammation [35,36], all of which are targets for researchers developing therapeutic agents. A particular target for treatment is the neuroinflammation induced mainly by activated microglia adjacent to the ischemic brain damage where it results in the production of neurotoxic molecules, likely proinflammatory cytokines or reactive molecules [17,36]. Part of therapeutic strategies against cerebral ischemia is focused on how to modulate the harmful functions of activated microglia which has been extensively studied, especially in herbal medicine field where many of these active molecules exert *in vivo* neuroprotective effects in cerebral ischemia [12,37,38]. In the current study, 6-paradol reduces neuroinflammatory responses in activated microglia, involving reduced productivities of NO, prostaglandins, and pro-inflammatory cytokines. The observed *in vitro* effects of 6-paradol on microglial activation were reaffirmed in a mouse model of cerebral ischemia, an M/R-challenged brain, where M/R-induced microglial activation was markedly reduced by the administration of 6-paradol, which ameliorated the ischemic brain damage. The 6-paradol’s efficacy on microglial responses remains even after 3 days following M/R challenge, which is obvious in periischemic regions where the penumbra lies. It would be noteworthy that most of therapeutic interventions have been developed to protect the ischemic penumbra region [39,40]. Therefore, the observed 6-paradol’s efficacy on microglial responses suggests that it may salvage the periischemic zone. In addition, the neuroprotective effect of 6-paradol was obvious when administered even after reperfusion, indicating that this compound possesses a therapeutic potential against cerebral ischemia.

The observed *in vivo* neuroprotection by 6-paradol is associated with the reduced expression of iNOS and TNF-α, both of which are well-known pathogenetic components in cerebral ischemia even though there is debate regarding the latter [41,42,43,44]. There are several cell types where these two neurotoxic molecules are upregulated or produced upon activated, which includes microglia, astrocytes, or infiltrated immune cells [45,46,47]. In this study, we also observed that 6-paradol reduced NO production, accompanied with the downregulation of iNOS expression, and TNF-α production in LPS-stimulated microglia. Therefore, the neuroprotective effects of 6-paradol in cerebral ischemia might be partly due to reducing expression levels of iNOS and TNF-α in microglia. It is still possible that neuroprotection could be from reduced production of those molecules in other cell types associated with neuroinflammation, such as reactive astrocytes or infiltrated immune cells. Nevertheless, the inhibitory effects of 6-paradol on iNOS and TNF-α can be applied to other many CNS disorders where these molecules are the main pathogenetic components, such as cerebral ischemia, multiple sclerosis, AD, PD, amyotrophic lateral sclerosis, or spinal cord injury [46,48]. In particular, the effect on TNF-α could be an important therapeutic potential because controlling TNF-α production would allow researchers to overcome the challenges of treating many of the previously mentioned CNS disorders [48].

Paradol, a non-pungent metabolite of shogaol by enzymatic reduction, is known to possess anti-inflammatory activities. Current *in vitro* findings demonstrate that the inhibitory properties of 6-paradol in treating neuroinflammation in microglia correlates to the *in vivo* therapeutic potential for cerebral ischemia. This study not merely provides evidence of 6-paradol’s neuroprotective efficacy in cerebral ischemia but also indicates its potential use in the treatment of other CNS disorders in which neuroinflammation is a pathological feature. This study may also explain the mechanism of action of 6-shogaol in diverse CNS disorders as it related to the biotransformation of 6-shogaol. In addition, if 6-paradol is shown to be effective in other CNS disorders, its non-pungent property has the advantage of fewer side effects on the stomach, which means it can be taken long-term, unlike that of ginger or ginger’s components likely 6-shogaol.

## Supporting Information

S1 Fig Effects of 2-, 4-, 6-, 8-, or 10-paradol on NO production and cell viability in LPS-stimulated BV2 microglia. Cells were pretreated with 10 μg/ml of paradol derivatives (2- to 10-paradol; 2P to 10P) or 6-shogaol (6S, 10 μg/ml) for 30 min and stimulated with 100 ng/ml LPS for 24 h. *p<0.05, **p<0.01, and ***p<0.001, versus cells treated with LPS alone. ^#^p<0.05, versus cells pretreated with 10 μg/ml of 6-paradol (6P) followed by LPS exposure. n = 3 per group.
(TIF)

S2 Fig Effects of 6-paradol on cell apoptosis in LPS-stimulated BV2 microglia. An Annexin V/propidium iodide (PI) apoptosis kit (Invitrogen) was used to quantify the percentage of cells undergoing apoptosis according to the manufacturer's instructions. The treated BV2 cells were washed twice with cold PBS, resuspended in binding buffer at a concentration of 1×10^6^ cells/μL, and stained with 5 μL of Annexin V‑FITC and 10 μL PI for 15 min at room temperature. The stained cells were analyzed immediately with FACS analysis system (FACSAriaIII; BD Biosciences, Franklin Lakes, NJ, USA). Data were collected from 10,000 events and analyzed using the Cell Quest software (BD Biosciences). The entire procedure was repeated three times for each sample. (A) Representative FACS data. X axis, Annexin V; Y axis, PI. (B) The percentages indicate the proportion of apoptotic cells in LPS group. Annexin V^+^/PI^-^cells, apoptotic cells; Annexin V^-^/PI^+^- cells, necrotic cells. ***p < 0.001, versus cells treated with LPS alone. n = 3 per group.
(TIF)

S3 Fig Effects of 6-shogaol and 6-paradol on brain injury in M/R-challenged mice. Mice were challenged with M/R and 6-shogaol (10 mg/kg, p.o.) or 6-paradol (10 mg/kg, p.o.) was administered immediately after reperfusion. Brain damages were assessed 22 h after reperfusion. (A-C) Effects of 6-paradol or 6-shogaol on infarct volume (A, B) and neurological function (C) were determined. Representative images of TTC-stained brain tissue (A) and quantification of brain infarction (B). Neurological score indicating neurological functions (C). M/R+veh, n = 6; M/R+6-paradol (6P), n = 6; M/R+6-shogaol (6S), n = 5. *p<0.05, versus vehicle-administered M/R mice (M/R+veh).
(TIF)

S4 Fig Administration of 6-paradol reduces microglial proliferation in the post-ischemic brain 3 days after M/R challenge. Mice were challenged with M/R and 6-paradol (10 mg/kg, p.o.) was administered immediately after reperfusion. Microglial proliferation was assessed 3 days after reperfusion by double immunolabeling using antibodies against Iba1 and BrdU. Representative low magnification images of BrdU-immunopositive cells. Scale bars, 400 μm.
(TIF)

S1 Table Primer sequences used for qRT-PCR analysis. (TIF)
