# Supplementary figures and images for: Neuroprotective Effect of 6-Paradol in Focal Cerebral Ischemia Involves the Attenuation of Neuroinflammatory Responses in Activated Microglia
Source: PLoS One. 2015 Mar 19;10(3):e0120203. doi: 10.1371/journal.pone.0120203 (PMC4366308; doi:10.1371/journal.pone.0120203)

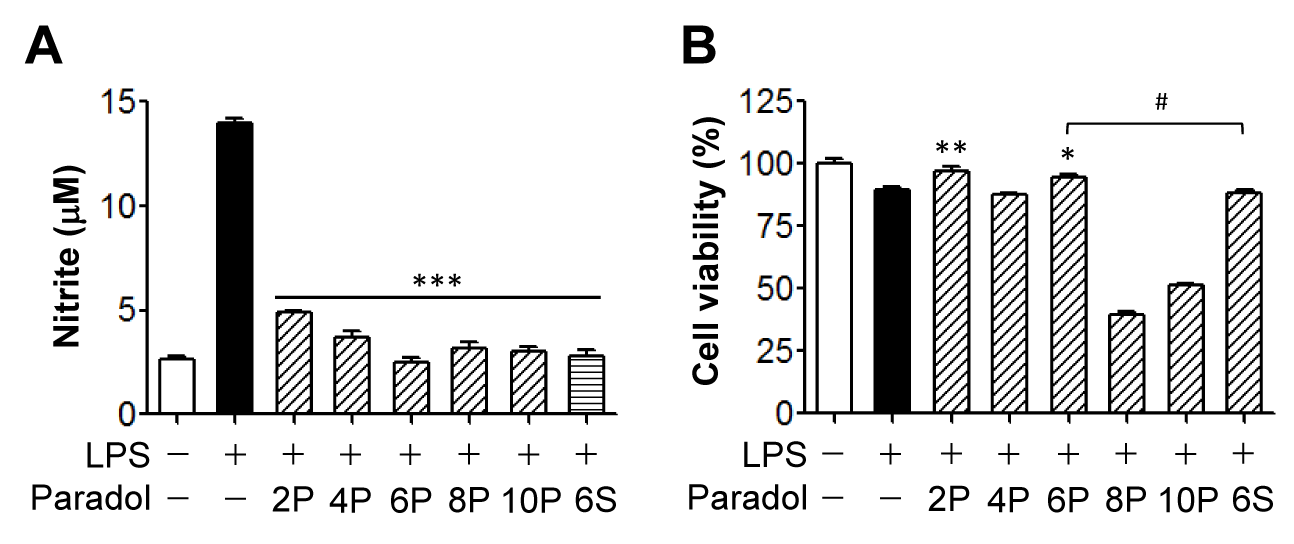

Supplement: S1 Fig — Cells were pretreated with 10 μg/ml of paradol derivatives (2- to 10-paradol; 2P to 10P) or 6-shogaol (6S, 10 μg/ml) for 30 min and stimulated with 100 ng/ml LPS for 24 h. *p<0.05, **p<0.01, and ***p<0.001, versus cells treated with LPS alone. #p<0.05, versus cells pretreated with 10 μg/ml of 6-paradol (6P) followed by LPS exposure. n = 3 per group. (TIF) [file pone.0120203.s001.tif]

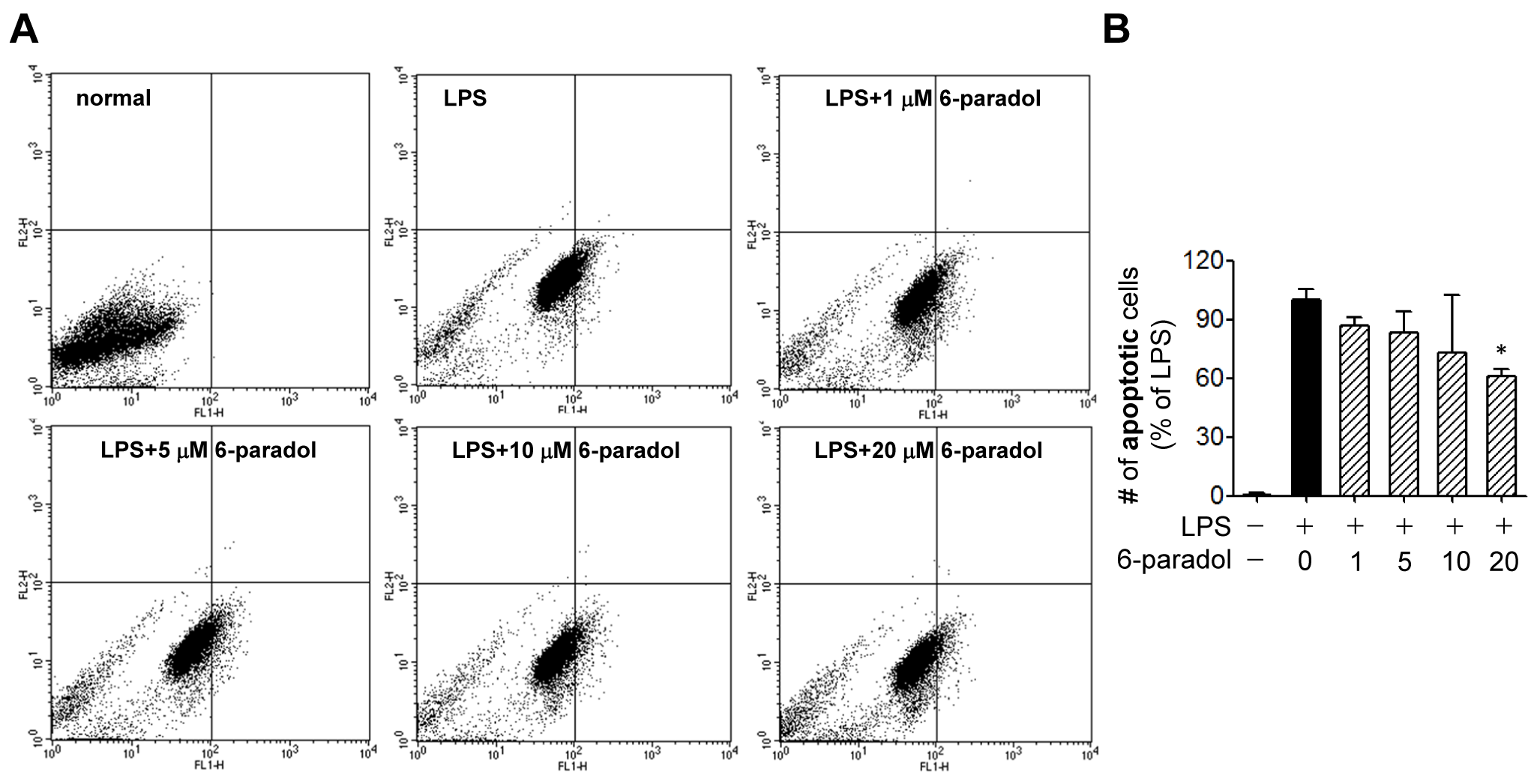

Supplement: S2 Fig — An Annexin V/propidium iodide (PI) apoptosis kit (Invitrogen) was used to quantify the percentage of cells undergoing apoptosis according to the manufacturer's instructions. The treated BV2 cells were washed twice with cold PBS, resuspended in binding buffer at a concentration of 1×10^6^ cells/μL, and stained with 5 μL of Annexin V‑FITC and 10 μL PI for 15 min at room temperature. The stained cells were analyzed immediately with FACS analysis system (FACSAriaIII; BD Biosciences, Franklin Lakes, NJ, USA). Data were collected from 10,000 events and analyzed using the Cell Quest software (BD Biosciences). The entire procedure was repeated three times for each sample. (A) Representative FACS data. X axis, Annexin V; Y axis, PI. (B) The percentages indicate the proportion of apoptotic cells in LPS group. Annexin V+/PI-cells, apoptotic cells; Annexin V-/PI+- cells, necrotic cells. ***p < 0.001, versus cells treated with LPS alone. n = 3 per group. (TIF) [file pone.0120203.s002.tif]

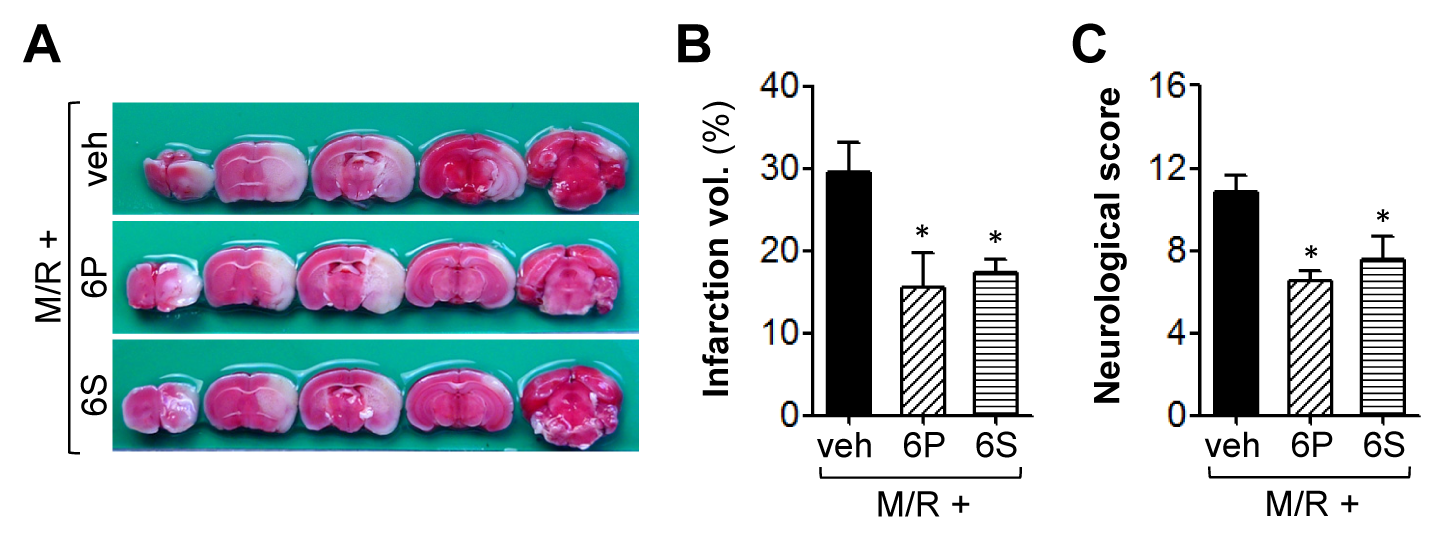

Supplement: S3 Fig — Mice were challenged with M/R and 6-shogaol (10 mg/kg, p.o.) or 6-paradol (10 mg/kg, p.o.) was administered immediately after reperfusion. Brain damages were assessed 22 h after reperfusion. (A-C) Effects of 6-paradol or 6-shogaol on infarct volume (A, B) and neurological function (C) were determined. Representative images of TTC-stained brain tissue (A) and quantification of brain infarction (B). Neurological score indicating neurological functions (C). M/R+veh, n = 6; M/R+6-paradol (6P), n = 6; M/R+6-shogaol (6S), n = 5. *p<0.05, versus vehicle-administered M/R mice (M/R+veh). (TIF) [file pone.0120203.s003.tif]

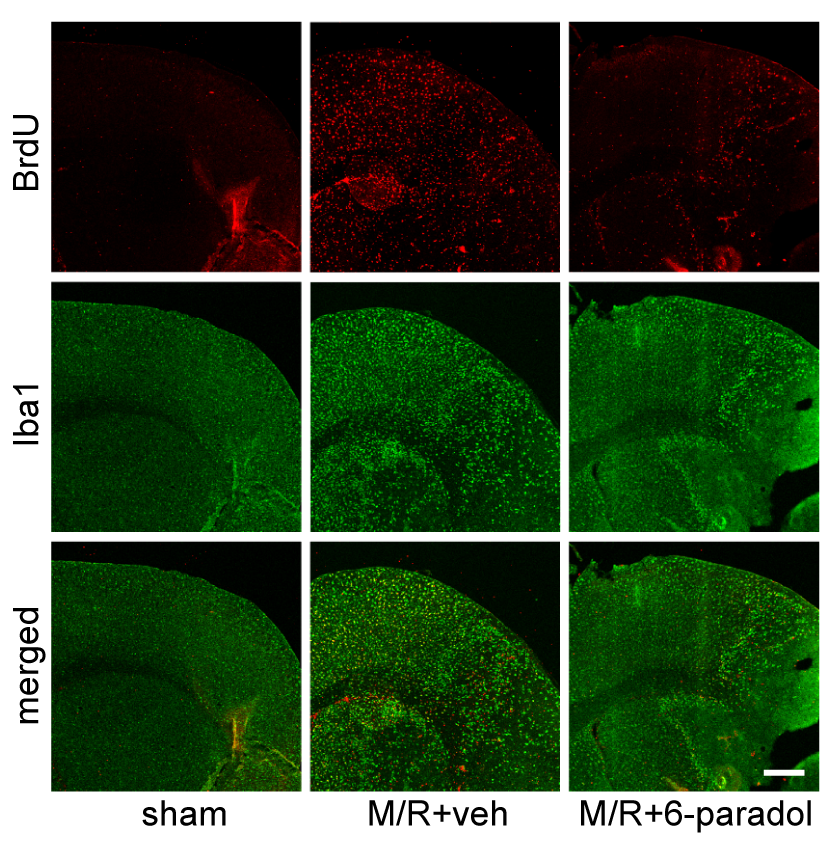

Supplement: S4 Fig — Mice were challenged with M/R and 6-paradol (10 mg/kg, p.o.) was administered immediately after reperfusion. Microglial proliferation was assessed 3 days after reperfusion by double immunolabeling using antibodies against Iba1 and BrdU. Representative low magnification images of BrdU-immunopositive cells. Scale bars, 400 μm. (TIF) [file pone.0120203.s004.tif]

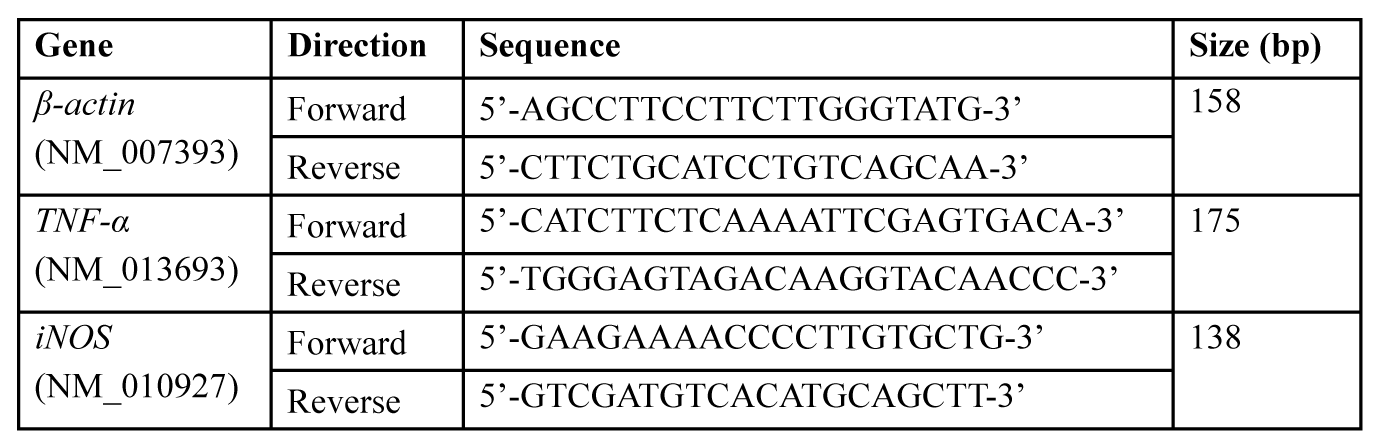

Supplement: S1 Table — (TIF) [file pone.0120203.s005.tif]
